# Institutional Satisfaction and Anxiety Mediate the Relationship Between Social Support and Depression in Hypertension Patients in Elderly Caring Social Organizations: A Cross-Sectional Study

**DOI:** 10.3389/fpsyg.2021.772092

**Published:** 2021-10-25

**Authors:** Kai Ji, Zhongliang Bai, Ling Tang, Huosheng Yan, Ying Zhu, Guimei Chen, Ren Chen

**Affiliations:** ^1^School of Health Services Management, Anhui Medical University, Hefei, China; ^2^Office of Science and Education, Suzhou Hospital Affiliated to Anhui Medical University, Suzhou, China

**Keywords:** social support, satisfaction, anxiety, depression, hypertension, elderly caring, social organizations, China

## Abstract

**Background:** Depression is a prevalent health condition among hypertension patients in elderly caring social organizations (SOs). Patients with hypertension and depression symptoms have worse health outcomes than those without depression. As the population ages, chronic and mental health issues such as depression of hypertension patients in elderly caring SOs have become prominent. However, the combined effects of social support, institutional satisfaction, and anxiety on depression among hypertension individuals in elderly caring SOs remain unclear. This study aimed to explore the mediating effects of institutional satisfaction and anxiety on the relationship between social support and depression among hypertension patients in elderly caring SOs in Anhui Province, China.

**Methods:** A cross-sectional study was conducted using a multi-stage stratified random sampling method. A questionnaire was used to collect data on demographic characteristics, the satisfaction of elderly caring SOs, social support, anxiety, and depression. A multiple linear regression model was utilized to investigate depression-related factors, and structural equation modeling (SEM) was employed to examine the relationships between social support, institutional satisfaction, anxiety, and depression among patients with hypertension in elderly caring SOs.

**Results:** Our results indicated that the mean scores of social support were 20.19 ± 6.98 and 1.92 ± 3.18 for anxiety, and 6.24 ± 5.03 for depression; besides, 33.3% of participants were very satisfied with elderly caring SOs, 48.5% were satisfied, and only 6.0% were dissatisfied or very dissatisfied. Comorbid chronic diseases were significantly associated with depression. Institutional satisfaction was directly negatively related to depression, whereas anxiety was directly positively correlated with depression. Social support had an indirect negative association with depression by the mediating effects of institutional satisfaction and anxiety.

**Conclusions:** The study highlights the importance of social support in maintaining mental health among hypertension patients residing in elderly caring SOs. To alleviate depression among hypertension patients in elderly caring SOs, strategies that target enhancing social support, institutional satisfaction, and anxiety reduction should be prioritized. More importantly, more attention should be paid to patients with comorbid chronic diseases.

## Introduction

Hypertension is one of the most common chronic diseases worldwide, accounting for two-thirds of all strokes and half of all coronary disease, and thus representing a major risk factor for cardiovascular morbidity and mortality (Chobanian et al., 2003; Perkovic et al., 2007; Prince et al., 2015). Numerous surveys conducted in China have revealed an increased prevalence of hypertension, affecting 18.8% of the population in 2002 and 27.8% in 2014 (Wu et al., 2008; Li et al., 2017). By 2025, this disease is anticipated to affect 29% of the world's population (Mittal and Singh, 2010). Due to the rapidly increasing prevalence of hypertension and disease burden, its effective and timely management and control have become a basic public health service priority in China.

Depression is the major cause of disability among patients with chronic diseases, impacting 350 million people worldwide (Davidson et al., 2000; Long et al., 2015). Hypertension patients are more likely to develop depression, and depression has been recognized as an independent risk factor for hypertension (Davidson et al., 2000; Meurs et al., 2015). Patients with hypertension and depression have worse health status, poorer quality of life, impaired well-being, higher health care expenditure, and increased mortality than those without depression (Oganov et al., 2011; Scuteri et al., 2011; Tsartsalis et al., 2016; Shao et al., 2017). Furthermore, patients with depression display lower adherence to hypertension treatment (Krousel-Wood and Frohlich, 2010), resulting in a 50% failure rate (Stephenson, 1999; Naderi et al., 2012). These studies highlight the importance of addressing depression in hypertension patients.

Social support is defined as “the social resources that persons perceive to be available or that are provided to them” (Gottlieb and Bergen, 2010). It is one of the most well-documented psychosocial factors associated with physical health outcomes (Cohen et al., 2000; Pinquart and Duberstein, 2010; Compare et al., 2013), including hypertension (Strogatz and James, 1986). Prior studies have demonstrated the association between social support and depression among patients with hypertension (Dennis et al., 2008; Ma, 2018). Poor social support has also been linked to poor adherence to anti-hypertension treatment and poor blood pressure control (Berkman et al., 2000; Taher et al., 2014; Ojo et al., 2016) and may thus cause poor prognosis and eventually influence the mental state of hypertension patients.

According to a previous report, anxiety was one of the most prevalent classes of problems in China and abroad, with ~5.3% of the population suffering from anxiety (Yu et al., 2018; Shi et al., 2020). Moreover, hypertension patients are at greater risk of exhibiting anxious symptoms (Cheung et al., 2005; Wei and Wang, 2006). Additionally, 39% of individuals with a generalized anxiety disorder have been demonstrated to meet depression criteria (Hunt et al., 2002).

Institutional satisfaction refers to the elderly's subjective evaluation of their overall satisfaction level, including quality of life, quality of care, and quality of service in elderly caring social organizations (SOs). Satisfaction with primary healthcare services appears to affect depression (Kavalniene et al., 2018). Numerous research has demonstrated a negative correlation between life satisfaction and depression (Gómez-Restrepo et al., 2013; Atienza-González et al., 2020; Köttl et al., 2021). However, the relationship between institutional satisfaction and depression among hypertension patients in elderly caring SOs remains unknown.

Hypertension is prevalent among nursing home residents, with prevalence ranging from 72 to 90% worldwide (Simonson et al., 2011; Könner et al., 2014; Benetos et al., 2015; Harris-Kojetin et al., 2016). The ineffective control of hypertension patients may increase their difficulty in coping with depressive symptoms and the possibility of developing anxiety and a low level of institutional satisfaction. In China, nursing homes and elderly apartments are classified into three major types, which can be operated by government organizations, SOs, or private investors (Chu and Chi, 2008). Commonly, the government-sponsored or -owned nursing homes are mainly served older people who have been disabled in financial resources. On the other hand, older people who are affordable are often covered by nursing homes that are operated by private investors. While the others have dwelled in nursing homes operated by SOs, which can play a major role in planning and developing relevant services for the elderly (Brick and Clarfield, 2007; Yang, 2018). Yet, little attention has been paid to the potential of SOs to participate in China's elderly care services.

Furthermore, previous studies had investigated associations between social support, institutional satisfaction, anxiety, and depression (Seabrook et al., 2016; Alipour et al., 2019; Barnett et al., 2019); however, the combined effects of these factors on depression and the underlying mechanisms of those relationships remain unclear. Besides, lacking social support has been positively associated with anxiety in type 2 diabetic patients (Wu et al., 2013) and hospitalized cardiac patients (Hughes et al., 2004). Given recent findings indicating that anxiety predicts depression (Gay et al., 2010, 2017) and the acknowledged role of social support in mental health (Yasin and Dzulkifli, 2010), we speculated that anxiety mediates the relationship between social support and depression in hypertension patients in elderly caring SOs. In addition, a prior study demonstrated the relationship between social support and life satisfaction, as well as their interactions in explaining depression among the elderly (Moon, 2010; Nam and Bok, 2013; Matud et al., 2019; Fang et al., 2021). Thus, we hypothesized the mediation effect of institutional satisfaction on the association between social support and depression among hypertension patients in elderly caring SOs. Moreover, researchers suggest that a high level of anxiety is associated with lower levels of life satisfaction (Rogowska et al., 2020). A low level of institutional satisfaction may cause hypertension patients to have feelings of distrust and disgust of elderly caring SOs, resulting in anxiety symptoms.

**Figure 1:**
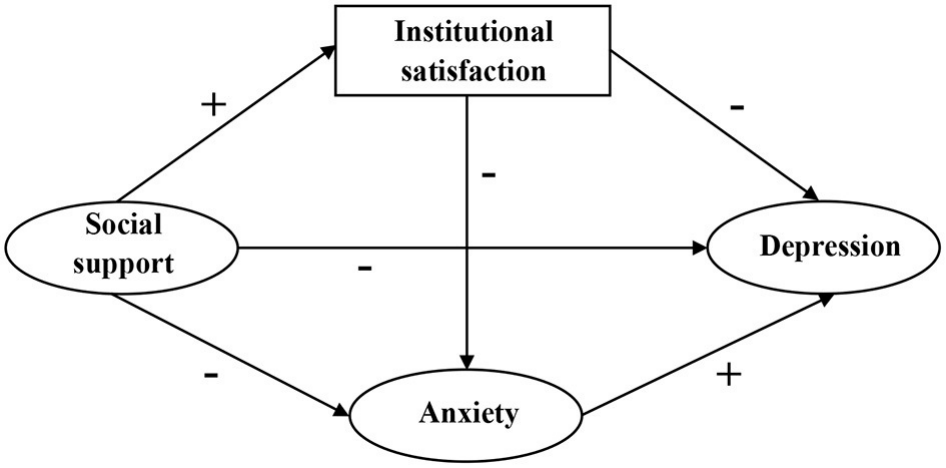
Theoretical model and hypotheses.

Thereby, to testify above mentioned research gaps and hypotheses, we employed structural equation modeling (SEM) to explore interrelationships between latent variables, which cannot be directly measured. According to the theoretical framework mentioned above, we developed the hypotheses shown in Table 1, corresponding to the structural equation model displayed in Figure 1. Eventually, we aimed to provide a reference for interventions targeting mental health improvement of hypertension patients in elderly caring social organizations.

**Table 1:** The theoretical hypotheses.

**Theoretical hypotheses**
1. Social support has a direct negative relationship with depression
2. Institutional satisfaction has a direct negative relationship with depression
3. Anxiety has a direct positive relationship with depression
4. Social support has a direct positive relationship with institutional satisfaction
5. Social support has a direct negative relationship with anxiety
6. Institutional satisfaction has a direct negative relationship with anxiety
7. The relationship between social support and depression is mediated by institutional satisfaction
8. The relationship between social support and depression is mediated by anxiety
9. The relationship between social support and anxiety is mediated by institutional satisfaction
10. The relationship between institutional satisfaction and depression is mediated by anxiety

## Materials and Methods

### Study Design and Data Collection

We conducted this cross-sectional survey in Anhui province, eastern China, between November and December 2019. Moreover, we used a multi-stage stratified random sampling method to ensure a representative sample considering socioeconomic levels and geographic location. As a result, we selected 15 elderly caring SOs from six cities in Anhui province: Fuyang, Anqing, Chizhou, Huainan, Luan, and Suzhou.

Following that, we identified potential participants, individuals diagnosed with hypertension in secondary hospitals and above, using chronic disease-related information from health records of residents in elderly caring SOs. With assistance from elderly caring SOs managers, skilled or trained graduate students personally conducted structured face-to-face interviews with participants. First, students verbally explained the study's purposes and procedures to interviewees, who were then requested to complete voluntary consent forms before the interviews. Those who could not fully understand verbal explanations due to severe deafness and limited communication skills were excluded. The analysis included 518 hypertension patients who agreed to be interviewed.

### Measures

#### Demographic Data

Demographic data comprised age, gender, education level, visited frequency of relatives, marital status, self-perceived health level, and comorbid chronic diseases. Age was categorized as <70, 70–79, and ≥80 years. The educational level was divided into middle school and above, primary school, and no formal education. The visited frequency of relatives was classified into three categories: <2, 2–4, and ≥5 (times per month). Marital status was defined as a binary variable: married with spouse and divorced, unmarried, or widowed. Self-perceived health level was divided into low, medium, and high. We asked participants whether they were diagnosed with heart disease, malignant tumors, chronic obstructive pulmonary disease, diabetes (type 1 or 2), neurological disorders, hyperlipidemia, chronic hepatitis, or other chronic diseases. Finally, hypertension patients were categorized into a comorbid chronic diseases group if they had at least one of the above-mentioned diseases (Yan et al., 2019).

#### Social Support

Social support was assessed using Multidimensional Scale of Perceived Social Support (MSPSS) assessment scores. Gregory developed MSPSS scale to identify the participants' perceived social support elements (Zimet et al., 1988). This measure includes three subscales assessing perceived support quality from family, friends, and special persons. Participants rated on a seven-point Likert response format (1 = “very strongly disagree” to 7 = “very strongly agree”). As a result, higher scores indicate high social support (Zimet et al., 1988). Scores from 12 to 48 indicate low social support, scores from 49 to 68 indicate moderate social support, and scores from 69 to 84 indicate high social support. In this study, Cronbach's α was found to be 0.98 for the family support subfield, 0.98 for the friend support subfield, 0.97 for the special person support subfield, and 0.96 for the scale in total, respectively, suggesting an adequate psychometric property in this sample.

#### Anxiety

Anxiety was measured using Generalized Anxiety Disorder 7-item Scale (GAD-7) (Spitzer et al., 2006). Participants indicate how frequently they have been bothered by each symptom over the last 2 weeks on a four-point Likert scale (0 = not at all, to 3 = nearly every day). Possible scores range from 0 to 21, with higher scores indicating higher levels of generalized anxiety. According to the scoring standard, the GAD-7 score is divided into 4 groups: 0–5, 6–10, 10–15, 16–21, corresponding to no, mild, moderate, and severe anxiety, respectively (Spitzer et al., 2006; Yang et al., 2020). GAD-7 has been shown to produce reliable and valid scores in community studies (Hinz et al., 2017), and the reliability in the current sample was acceptable (Cronbach's α = 0.88).

#### Institutional Satisfaction

A single item, “What is your overall satisfaction with the elderly caring social organizations?” was used to measure the institutional satisfaction of hypertension patients in elderly caring SOs. Many studies use a single item to measure overall satisfaction in large-scale satisfaction surveys with good reliability (Labarbera and Mazursky, 1983; Yi, 1990; Mittal et al., 1998). In this study, we employed a single item with a Likert 5-point option to measure the overall satisfaction of elderly caring SOs, where 1 represents very dissatisfied, and 5 represents very satisfied.

#### Depression

To assess depression, the Center for Epidemiologic Studies Depression Scale 10-item version (CESD-10) was employed, which has been demonstrated to appropriately reflect depressive symptoms experienced in the previous week (Andresen et al., 1994). CESD-10 includes ten items addressing depressed affect, somatic symptoms, and positive affect. The options for each item range from “rarely or none of the time” (score of 0) to “all of the time” (score of 3). The scoring is reversed for items 5 and 8, which are positive effect statements. Total scores can range from 0 to 30. Scores of 10 or over indicate clinically relevant depression (Andresen et al., 1994). The scale has excellent internal reliability (Cronbach's α = 0.80) and good validity (Roberts and Vernon, 1983). In the present study, Cronbach's α coefficient was acceptable (Cronbach α =0.81).

### Statistical Analysis

Statistical analyses were conducted using SPSS 23.0 and MPLUS 8.3. First, descriptive statistics were calculated to describe the sample, and continuous variables are presented as mean ± standard deviation, while categorical variables are presented as percentages (%). Second, Pearson correlations were employed to explore the relationships between social support, institutional satisfaction, anxiety, and depression. A multiple linear regression model was used to estimate associations between independent variables and depression. Finally, SEM was used to test the hypotheses about four study variables. We used subscale scores of social support, scale scores of anxiety and depression as measurement variables, then used the total scores of these measures as latent variables. Institutional satisfaction was included as a measurement variable. Statistical significance was set at *P* < 0.05.

## Results

### Descriptive Analysis

Descriptive statistics of the sample are displayed in Table 2. The participants contained 298 men (57.5%) and 220 women (42.5%). The highest proportion was 70–79 years old (42.7%), with no formal education (51.9%). Most participants were divorced, widowed, or unmarried (82.0%) and had comorbid chronic diseases (53.7%). A large proportion of hypertension patients in elderly caring SOs are visited by relatives less than twice a month (43.2%), and low levels of self-perceived health (30.7%) are more than those high levels of self-perceived health (13.7%).

**Table 2:** Descriptive results of the sample.

**Variables**	***N*** **(%), Mean ± SD**
**Gender**	
Male	298 (57.5)
Female	220 (42.5)
**Age (years)**	
<70	112 (21.7)
70–79	221 (42.7)
≥80	185 (35.6)
**Education**	
No formal education	269 (51.9)
Primary school	140 (27.0)
Middle school and above	109 (21.1)
**Marital status**	
Married have spouses	93 (18.0)
Divorced, widowed, or unmarried	425 (82.0)
**Visited frequency of relatives (times/month)**	
<2	224 (43.2)
2–4	96 (18.5)
≥5	198 (38.3)
**Comorbid chronic diseases**	
No	240 (46.3)
Yes	278 (53.7)
**Self-perceived health level**	
Low	159 (30.7)
Medium	288 (55.6)
High	71 (13.7)
**Institutional satisfaction**	
Very dissatisfied	1 (0.2)
Dissatisfied	30 (5.8)
General	63 (12.2)
Satisfied	251 (48.5)
Very satisfied	173 (33.3)
**Social support**	60.69 ± 16.42
From family	20.19 ± 6.98
From friends	19.80 ± 5.96
From significant others	20.69 ± 5.78
**Anxiety**	1.92 ± 3.18
**Depression**	6.24 ± 5.03

*Continuous variables are presented as mean ± standard deviation; categorical variables are presented as number (%)*.

The mean scores for social support, anxiety and depression were 60.69 ± 16.42, 1.92 ± 3.18, and 6.24 ± 5.03, respectively. The proportion of individuals with a high level of social support was 38.0%, and 86.7% of subjects had no anxiety symptoms. In addition, 21.4% of hypertension patients in elderly caring SOs exhibited depressive symptoms. Furthermore, 81.8% of participants were satisfied or very satisfied with their elderly caring SOs.

#### Correlations Between Study Variables

Correlations between social support, institutional satisfaction, anxiety, and depression are presented in Table 3. Social support was negatively correlated with anxiety and depression but positively correlated with the satisfaction of elderly caring SOs. Additionally, there was a significant negative correlation between institutional satisfaction and anxiety and depression. Anxiety was significantly positively correlated with depression.

**Table 3:** The correlation among key variables.

**Variables**	**Social support**	**Institutional satisfaction**	**Anxiety**	**Depression**
Social support				
Institutional satisfaction	0.335**			
Anxiety	−0.164**	−0.208**		
Depression	−0.296**	−0.378**	0.658**	

** *P < 0.001*.

#### Linear Regression Analysis of Study Variables

Table 4 reveals that depression among hypertension patients in elderly caring SOs was associated with the factor of comorbid chronic diseases, in addition to social support, institutional satisfaction, and anxiety. The depressive symptoms of hypertension patients who had comorbid chronic diseases were more serious than those who did not have (β = 0.105, *P* = 0.001).

**Table 4:** Multiple linear regression analysis of factors associated with depression.

	**B**	**t**	***P***	**95% CI**
Constant	5.444	5.670	<0.001	3.558, 7.331
**Gender**				
Male (ref)				
Female	0.571	1.589	0.113	−0.135, 1.276
**Age (years)**				
<70 (ref)				
70–79	−0.043	−0.106	0.916	−0.851, 0.764
≥80	0.761	1.698	0.090	−0.120, 1.643
**Education**				
No formal education (ref)				
Primary school	−0.644	−1.702	0.089	−1.387, 0.099
Middle school and above	−0.281	−0.665	0.506	−1.111, 0.549
**Marital status**				
Married have spouses (ref)				
Divorced, widowed, or unmarried	0.011	0.027	0.978	−0.822, 0.845
**Visited frequency of relatives (times/month)**				
<1 (ref)				
1–5	−0.724	−1.559	0.120	−1.636, 0.189
≥5	0.447	0.997	0.319	−0.433, 1.326
**Comorbid chronic diseases**				
No (ref)				
Yes	1.075	3.341	0.001	0.443, 1.708
**Self-perceived health level**				
Low (ref)				
Medium	−0.108	−0.310	0.757	−0.794, 0.578
High	−0.513	−1.001	0.317	−1.521, 0.494
**Institutional satisfaction**				
Very satisfied (ref)				
Satisfied	1.366	3.777	<0.001	0.655, 2.076
General	2.975	5.447	<0.001	1.902, 4.048
Dissatisfied	3.357	4.731	<0.001	1.963, 4.751
Very dissatisfied	3.829	1.069	0.285	−3.206, 10.864
**Social support**	−0.048	−4.041	<0.001	−0.071, −0.025
**Anxiety**	0.894	17.191	<0.001	0.791, 0.996

#### Test of Study Model

**Figure 2:**
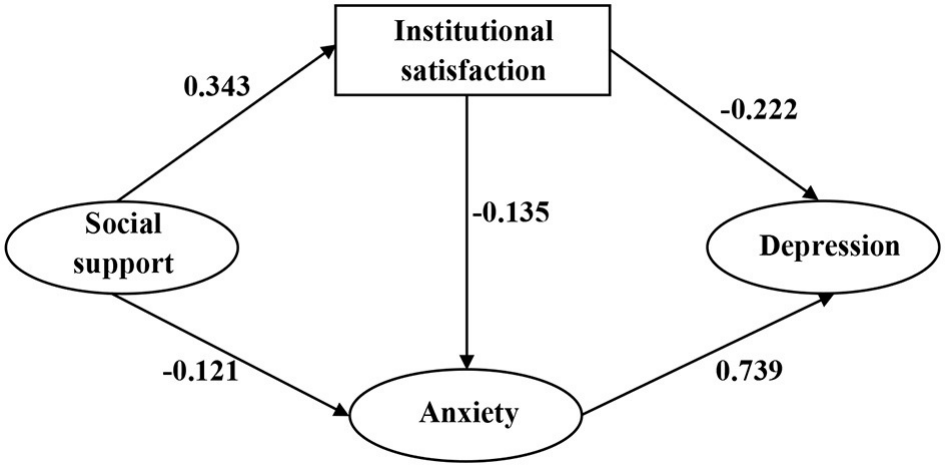
Structural analysis of social support, institutional satisfaction, anxiety and depression. All coefficients are significant (*P* < 0.05).

SEM was used to test the model depicted in Figure 1. The path coefficient of the link between social support and depression was not statistically significant. As a result, we revised our model by removing this path. After setting socio-demographic characteristics as covariates, the direction of influence among key variables remained unchanged, and corresponding coefficients remained unchanged. Thus, the socio-demographic characteristics were not confounding factors and were excluded from the final model. To improve model fitness, the covariance between measurement errors was set based on modification indices. Figure 2 displays the final modified model that examined the associations between depression and social support, institutional satisfaction, and anxiety. Standardized coefficients representing direct associations between variables are displayed over the arrows. The model demonstrated good fit: χ^2^ = 631.57 (*p* < 0.001), χ^2^/df = 3.53, CFI = 0.93, TLI = 0.92, SRMR = 0.06, RMSEA = 0.07.

The total, direct, and indirect effects between social support, institutional satisfaction, anxiety, and depression are displayed in Table 5. Institutional satisfaction directly affected the depression (β = −0.228, 95% CI: −0.302 to −0.155) of hypertension patients in elderly caring SOs, thus supporting Hypothesis 2. Anxiety was directly associated with depression (β = 0.745, 95% CI: 0.670–0.808), corroborating Hypothesis 3. However, social support was only indirectly associated with depression (β = −0.203, 95% CI: −0.289 to −0.124), rather than directly associated, leading us to reject Hypothesis 1. Greater social support was linked to an enhanced likelihood of having higher institutional satisfaction (β = 0.343, 95% CI: 0.250–0.435) and a lower level of anxiety (β = −0.122, 95% CI: −0.236 to −0.011), corroborating Hypotheses 4 and 5. Institutional satisfaction exhibited a direct association with the anxiety of hypertension patients (β = −0.134, 95% CI:−0.256 to−0.011), corroborating Hypothesis 6.

**Table 5:** Direct, indirect, and total effects of key study variables.

**Model pathways**	**Standardized coefficient**	**95% CI**
**Total effects**		
Social support → Institutional satisfaction	0.343	0.250 to 0.435
Social support → Anxiety	−0.168	−0.270 to −0.070
Social support → Depression	−0.203	−0.289 to −0.124
Institutional satisfaction → Anxiety	−0.134	−0.256 to −0.011
Institutional satisfaction → Depression	−0.328	−0.432 to −0.216
Anxiety → Depression	0.745	0.670 to 0.808
**Direct effects**		
Social support → Institutional satisfaction	0.343	0.250 to 0.435
Social support → Anxiety	−0.122	−0.236 to −0.011
Institutional satisfaction → Anxiety	−0.134	−0.256 to −0.011
Institutional satisfaction → Depression	−0.228	−0.302 to −0.155
Anxiety → Depression	0.745	0.670 to 0.808
**Indirect effects**		
Social support → Anxiety	−0.046	−0.098 to −0.003
Social support → Depression	−0.203	−0.289 to −0.124
Institutional satisfaction → Depression	−0.100	−0.189 to −0.008

Table 6 displays significance testing results of mediating pathways. A mediating effect was considered statistically significant if 95% confidence interval did not include zero. The results illustrated that institutional satisfaction and anxiety mediated the relationship between social support and depression (95% CI: −0.116 to −0.047 and −0.179 to −0.008, respectively), supporting Hypotheses 7 and 8. Besides, institutional satisfaction mediated the relationship between social support and anxiety (95% CI: −0.098 to −0.003), corroborating Hypothesis 9. Anxiety mediated the relationship between institutional satisfaction and depression (95% CI: −0.189 to −0.008), thus supporting Hypothesis 10.

**Table 6:** Significance of mediating pathways.

**Model pathways**	**95% CI**
Social support → Institutional satisfaction → Depression	−0.116 to −0.047
Social support → Anxiety → Depression	−0.179 to −0.008
Social support → Institutional satisfaction → Anxiety	−0.098 to −0.003
Institutional satisfaction → Anxiety → Depression	−0.189 to −0.008

## Discussion

To the best of our knowledge, this research is the first to explore the relationships between social support, institutional satisfaction, anxiety, and depression in hypertension patients in elderly caring SOs in China.

In the current study, lower institutional satisfaction was linked to an increased risk of depression in hypertension patients in elderly caring SOs. Lower institutional satisfaction may result in lower quality of life and trust, a weaker sense of happiness, and easy to produce negative emotions, which is similar to a study indicating a significant negative correlation between depression and life satisfaction in nursing home residents (Kim and Hwang, 2017). Additionally, high-level institutional satisfaction may be conducive to hypertension control and prognosis. A study found that the correlation between patient satisfaction and compliance to treatment is well documented (Calabro et al., 2018).

The present study found that anxiety was positively linked to depressive symptoms in study samples, consistent with previous research showing that anxiety was an independent predictor of depressive symptoms (Gay et al., 2017). Individuals with high anxiety levels often exhibit symptoms such as fear, worry, insomnia, and fatigue, leading to accumulation of negative emotions that cannot be resolved for a long time (Gay et al., 2017), which may contribute to depressive symptoms in this group. Interestingly, our study evidenced the mediating effect of anxiety on the relationship between institutional satisfaction and depression. Hypertension patients in elderly caring SOs with a high level of institutional satisfaction may have more trust in elderly caring SOs, promoting communication with others and improving interpersonal relationships, and ultimately reducing their risk of depression. In other words, anxiety not only has a direct positive relationship with depression but has also been shown to weaken the possible beneficial effects of institutional satisfaction on depression.

According to previous research, subjects with a lower level of social support are more likely to develop cardiovascular disease due to a history of hypertension. They are at increased risk of experiencing higher blood pressure, less nocturnal blood pressure decrease, and a worse prognosis after a cardiovascular event (Blumenthal et al., 1987; Rosengren et al., 2004; Barth et al., 2010). One study reported a threefold increase in the risk of all-cause mortality in hypertension patients with poor social support (Menéndez-Villalva et al., 2015). Consequently, insufficient social support offered to hypertension patients calls for more attention. In this study, participants received relatively little support from family and friends. Therefore, special attention needs to be given to hypertension patients in elderly caring SOs who lack the care of friends and family members and improve their social support to reduce negative emotions.

Multiple literature documented that lack of social support is a strong predictor of depression in hypertension patients (Dennis et al., 2008; Ma, 2018). However, different from other studies, our study indicated that the direct relationship between social support and depression was not significant in the study samples. Through the mediating effects of institutional satisfaction and anxiety, social support had an indirect effect on depression. A lack of social support was associated with a higher likelihood of having a low level of institutional satisfaction, suggesting that social support can increase the trust and sense of belonging in hypertension patients in elderly caring SOs, thereby enhancing institutional satisfaction should be emphasized. The mediating pathways revealed that lack of social support might impact the risk of institutional dissatisfaction and cause anxiety, thus potentially contributing to a higher prevalence of depression. This study contributes to our understanding of how social support affects depression and offers strategies that may benefit the mental health of hypertension patients.

Furthermore, linear regression revealed that comorbid chronic diseases were associated with depression among hypertension patients in elderly caring SOs. Participants with comorbid chronic diseases had a higher degree of depression than those without, which is consistent with previous studies (Findley et al., 2011; Ma, 2018). Higher physical comorbidities were associated with an increased risk of depression in hypertension patients in elderly caring SOs, resulting from persistent limitations in daily functioning associated with coexisting diseases.

Overall, relieving depressive symptoms in hypertension individuals in elderly caring SOs requires social support enhancement, institutional satisfaction, and anxiety reduction. In particular, more attention should be directed toward hypertension patients with comorbid chronic diseases in elderly caring SOs. More institution-based collective activities and social opportunities should be provided for hypertension individuals in elderly caring SOs to address the lack of social support caused by conditions. Moreover, care providers are an important social support resource whose activities in this regard should be encouraged (Chen et al., 2018). To enhance the institutional satisfaction of hypertension patients within elderly caring SOs, we suggest that elderly caring SOs should improve their environment and service quality while also expanding social capital into the elderly care sector to provide targeted elderly care services. The findings of previous study indicated that nursing home residents are more satisfied in smaller nursing homes and nursing homes with frequent opportunities for physical and social activity (Spangler et al., 2019). In addition, as artificial intelligence advances, applying data-driven intelligent platforms has huge potential in health management and health decision-making (Gu et al., 2019), effectively improving organizational performance and internal and external satisfaction. Elderly caring SOs should also pay attention to the anxiety of hypertension patients and take steps to reduce their anxiety levels.

This study has several limitations. First, the cross-sectional design of this study describes the relationships between depression and social support, anxiety, and institutional satisfaction; it does not enable one to infer causality of the three determinants on depression in hypertension individuals in elderly caring SOs. Further studies using a longitudinal or randomized control trial design are proposed. Second, since we conducted this study in Anhui Province, the generalizability of our findings is limited. Future studies that include expanded areas and larger sample size are required.

Despite the above limitations, our findings are reliable because the sample was representative and had high response rates. Furthermore, our study adds important findings regarding the factors that influence depression and mechanisms underlying these factors' relationships in hypertension patients in elderly caring SOs and provide insights into designing focused and effective measures for depression prevention in hypertension patients in elderly caring SOs and control work.

## Conclusion

Our findings indicated that social support, institutional satisfaction, anxiety, and comorbid chronic diseases were significantly associated with depression in hypertension patients in elderly caring SOs and elucidated possible mechanisms behind these variables. Institutional satisfaction had a direct negative correlation with depression, whereas anxiety was directly positively associated with depression. Social support was indirectly negatively associated with depression, mediated by anxiety and institutional satisfaction. Institutional satisfaction also had an indirect negative effect on depression *via* anxiety. In addition, hypertension patients with comorbid chronic diseases in elderly caring SOs exhibited more depressive symptoms.

## Data Availability Statement

The original contributions presented in the study are included in the article/supplementary materials, further inquiries can be directed to the corresponding author.

## Ethics Statement

This study was approved and ethical approval was obtained from the Biomedical Ethics Committee, Anhui Medical University (No. 20180181). The patients/participants provided their written informed consent to participate in this study.

## Author Contributions

KJ and ZB: conceptualization. HY and GC: methodology. YZ and LT: investigation. KJ: writing—original draft preparation. KJ, ZB, and RC: writing—review and editing. All authors have read and agreed to the published version of the manuscript.

## Funding

This study was funded by the National Nature Science Foundation of China (Nos. 71874002 and 72174001) and Research fund of Anhui Medical University (No. 2021xkj255).

## Conflict of Interest

The authors declare that the research was conducted in the absence of any commercial or financial relationships that could be construed as a potential conflict of interest.

## Publisher's Note

All claims expressed in this article are solely those of the authors and do not necessarily represent those of their affiliated organizations, or those of the publisher, the editors and the reviewers. Any product that may be evaluated in this article, or claim that may be made by its manufacturer, is not guaranteed or endorsed by the publisher.
